# Bioinoculants as a means of increasing crop tolerance to drought and phosphorus deficiency in legume-cereal intercropping systems

**DOI:** 10.1038/s42003-023-05399-5

**Published:** 2023-10-06

**Authors:** Bouchra Benmrid, Cherki Ghoulam, Youssef Zeroual, Lamfeddal Kouisni, Adnane Bargaz

**Affiliations:** 1 Plant-Microbe Interactions Laboratory, AgroBiosciences Program, College for Sustainable Agriculture and Environmental Sciences, Mohammed VI Polytechnic University, Ben Guerir, 43150 Morocco; 2 Agrobiotechnology & Bioengineering Center, Research Unit CNRST labeled, Cadi Ayyad University, Faculty of Sciences and Techniques, 40000 Marrakech, Morocco; 3 Situation Innovation – OCP Group, Jorf Lasfar, 24025 Morocco; 4 African Sustainable Agriculture Research Institute, Mohammed VI Polytechnic University, Laayoune, Morocco

**Keywords:** Soil microbiology, Abiotic, Drought

## Abstract

Ensuring plant resilience to drought and phosphorus (P) stresses is crucial to support global food security. The phytobiome, shaped by selective pressures, harbors stress-adapted microorganisms that confer host benefits like enhanced growth and stress tolerance. Intercropping systems also offer benefits through facilitative interactions, improving plant growth in water- and P-deficient soils. Application of microbial consortia can boost the benefits of intercropping, although questions remain about the establishment, persistence, and legacy effects within resident soil microbiomes. Understanding microbe- and plant-microbe dynamics in drought-prone soils is key. This review highlights the beneficial effects of rhizobacterial consortia-based inoculants in legume-cereal intercropping systems, discusses challenges, proposes a roadmap for development of P-solubilizing drought-adapted consortia, and identifies research gaps in crop-microbe interactions.

## Abstract (web summary)

This review discusses the intricate interactions between rhizobacterial consortia-based inoculants, cropping systems, and soil abiotic stresses, focusing specifically on drought and phosphorous deficiency.

## Introduction

Nowadays, feeding the growing population is becoming one of the world’s major concerns due to the ever-increasing need for agricultural products^1,2^. Water scarcity represents one of the serious threats that has emerged over the past few decades, deteriorating the whole plant-soil system^3,4^. In addition to drought, phosphorus (P) deficiency is viewed as one of the main nutrient limitations restraining worldwide crops growth and production owing to its irreplaceable role and its limited availability^5–8^. Drought and P-deficiency, either as individual or combined constraints, cause severe disruptions of the plants’ morphological, physiological, biochemical, and molecular processes^9–11^. In many regions of the world, the incidence and the extent of drought and P-deficiency, together, are expected to increase which will put more pressure on the agricultural sector as there is a direct relationship between soil water status, P movement, and plant growth and yield^12^.

The addition of P fertilizers is considered as an efficient strategy to compensate for P-deficiency and to stimulate plants’ growth under water-deficit conditions^6,13^. Although, being unquestionably needed for crop production, large amount of these water-soluble P fertilizers may be rendered unavailable for plants due to fixation by cations such as Al^3+^, Fe^3+^, Ca^2+^, clay particles, or transformed into organic forms, hence their efficacy is reduced^14,15^. Chemical P-fertilizers are manufactured based on rock phosphate (RP), a non-renewable P source that has been recently exploited for its direct use in high P-retention soils^16^. However, thorough investigations are still needed to increase the RP agronomic efficiency.

In this context, growing interest has been given to exploiting biological resources, notably, rhizosphere soil microorganisms as they represent a low-input, environmentally friendly biotechnology to improve P nutrition and enhance plant growth^17,18^. Among these, P-solubilizing bacteria (PSB) are soil microorganisms that can transform insoluble P into soluble P forms, thus making it available for plants^19–21^. More interestingly, many of the PSB were found able to enhance the plants’ tolerance to several abiotic stresses, encompassing drought^22^.

**Fig. 1:**
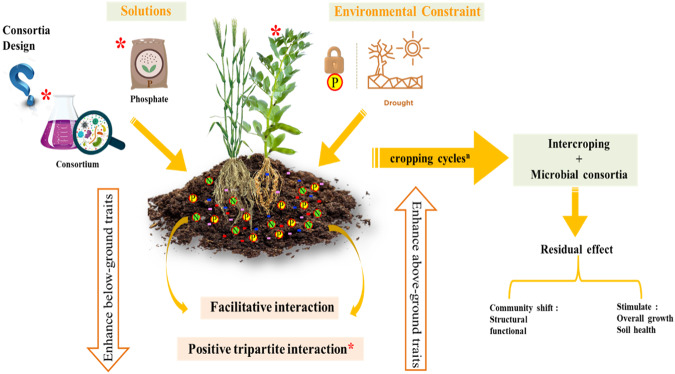
Drought-tolerant consortia and intercropping for resilient agriculture. Drought-tolerant P-solubilizing consortia, when combined with intercropping systems, have the potential to significantly improve plants’ resilience to abiotic stresses such as water scarcity. This synergistic approach may not only benefit the current crop cycle but also leave a lasting positive impact on subsequent cropping cycles. By enhancing nutrient availability and stress tolerance, these consortia offer a promising strategy for sustainable and resilient agricultural practices.

Rhizosphere dwellers are also known as the second plant genome as their contribution to plant growth promotion is likely synchronized by the host-plant itself^23^. Indeed, pioneering studies on the root-rhizobacteria interaction have focused on the effect of rhizo-deposits on plant-specific rhizo-microbiome synchronization^24–26^. The interrelationship between plant roots and beneficial rhizobacteria has been studied since the early 1980’s^27^. Ever since, greatest importance was given to decipher the interactional mechanisms that leads to successful and robust plants’ rhizosphere colonization by beneficial plant growth promoting rhizobacteria (PGPR). Rhizospheric microorganisms are generally endowed with a wide spectrum of plant growth promoting traits. Therefore, it is generally believed that inoculating drought and/ or P-stressed plants with microorganisms unifying various growth promoting traits can cooperate among themselves to give the highest positive results (Fig. 1). A good example of this, is the cooperative association between nodule endophytic bacteria, the so-called rhizobia, and other rhizosphere bacteria which have been found to allow better response to disturbances and adaptation to harsh environmental conditions^27^.

Furthermore, it was recently reported that among the different agricultural management practices, cropping systems, notably intercropping, represent a promising strategy to increase crops productivity, minimize fertilizers inputs, and ameliorate fertility in N and P-deficient soils^28–31^. For instance, legume-cereal intercropping systems particularly debated in this review, has been reported to support better growth of both intercrops under stressful environmental conditions^32,33^ (Table 1) (Fig. 1). Owing to their lower competition with cereals, legumes were reported to increase the N as well as P nutrition of cereals in low input soils, improving the availability of water resources, and increasing the land productivity^32–35^. The dynamic interaction within the soil microbial communities of intercropped plant species was also reported to be a major driver of interspecific facilitation in intercropping systems^28,32,36^. In fact, intercropped species may have some control over the shifts in the structural and functional composition of soil rhizobacteria^29,37,38^. As an example, bacterial and fungal diversity varied in intercropped, as compared to sole cropped, wheat and soybean species^32^.

**Table 1:** A non-exhaustive list of studies highlighting the positive interactions between microbial inoculants and intercropping systems on plant growth and stress tolerance.

Intercropped plants	Stressors	PGPR strains	Strains origin	Stress avoidance mechanisms	Growth promotion traits	Production system	Reference
Pigeon pea – maize	–	*Enterobacter* sp. C1D *Pseudomonas* sp. G22 *Rhizobium* sp. IC3109	Sediment sample at an industrial waste effluent Groundnut rhizosphere Pigeon pea nodules	–	P-solubilization, N-fixation Cd and Cr-tolerance, ACC-deaminase, IAA, and siderophore, DAPG production	Greenhouse experiment	^27^
Faba bean – maize	P-deficiency Desert soil	*Rhizobium leguminosarum*	Faba bean nodules	Interspecific facilitation in *Rhizobium-faba* bean-maize intercropping system conferred advantage in nodulation, facilitated P nutrition in desert soil	Symbiotic N-fixation	Field experiment	^148^
Faba bean – durum wheat	P-deficiency	*Rahnella aquatilis* *Pseudomonas sp*	Rhizosphere soil of wheat and faba bean	Co-inoculation with PGPR along with intercropping enhanced the bioavailability of nutrients notably N and P, eventually improving plant growth	P and K solubilization, N-fixation, exopolysaccharides, IAA and siderophores production	Greenhouse experiment	^39^
Fenugreek – barley	Low-rainfall soils	*Sinorhizobium meliloti* F42 *Variovorax paradoxus* F310	Fenugreek nodules	Inoculation improved fenugreek nodulation parameters, increased P and N bioavailability which all enhanced growth parameters	P solubilization, symbiotic N-fixation, IAA, ACC, HCN and siderophore production	Greenhouse and field experiments	^58^
Finger millet – pigeon pea	Drought stress	*Rhizophagus irregularis* *Pseudomonas fluorescens* *Bradyrhizobium* sp.	Wheat roots Pigeon pea nodules	The shallow-rooted finger millet profited from intercropped deep- rooted pigeon pea as it has access to a bottom wet soil layer,allowing better water and mineral nutrients uptake. AMF and PGPR also increased N and P uptake by both plants under drought-conditions	P-solubilization, symbiotic N-fixation, and production of IAA, siderophores, ACC-deaminase, and diacetyl-phloroglucinol	Greenhouse experiment	^59^
Soybean – maize	–	*Rhizophagus irregularis* *Streptomyces* sp. *Bacillus megaterium*	AMF spores	Synergistic use of AMF, PGPR, and intercropping induced greatest plant growth reponses and highest P-uptake, at 50% of the regular recommended P rate for maize-soybean intercropping.	P-solubilization/mineralization (phytate), siderophore and IAA production	Greenhouse experiment	^31^
Red-clover – ryegrass	P-deficiency	*Rhizobium* sp. T88 *Herbaspirillum* sp. AP21	–	Inoculants induced beneficial effect on plants via increment of rock P use efficiency and growth promotion, which are associated with their phenotypic and genomic characteristics related with P solubilization and mineralization.	P-Solubilization (RP and TCP)/ mineralization (phytate), symbiotic N-fixation, IAA and exopolysaccharides production	Greenhouse experiment	^149^

Meanwhile, application of microbial consortia has been thoroughly deciphered at both fundamental and applied levels, and the benefits of these microorganisms on plant growth under harsh environmental conditions are well-documented. However, only few studies have reported the effect of microbial consortia on the growth of intercropped cereals and leguminous plants^27,39^. Yet, one of the main factors hindering the predictability and effective management of microbial inoculants for different cropping systems is the soil abiotic factors to which the response of microbial inoculants remained unstable and sometimes, inefficient. Therefore, it would be worthwhile to identify constraints associated with in-field application of microbial consortia, particularly in intercropping systems under drought and/or P-deficiency.

In this review, shortcomings of the intricate interaction between microbial inoculants, cropping systems, and soil abiotic stresses - with focus on drought and P-deficiency – will be discussed. Therefore, first, (i) the effects of drought and P-deficiency on the plant-soil-microbe system will be highlighted. Then, the significance of shifts in resident microbial composition in response to cropping patterns, drought, and P-deficiency, as well as their potential to influence soil ecosystem processes, will be examined.

In the second part, (ii) a comprehensive knowledge of the interrelationship between plant roots and the beneficial rhizobacteria communities is provided, at both fundamental and applied levels. The insights into these beneficial effects are based on a detailed presentation of relevant examples highlighting particularly at the applied level the ecological significance of drought-tolerant phosphate solubilizing rhizobacteria, with specific focus on multi-strains microbial inoculants as an essential component of the plant-soil system. Afterwards, (iii) the emerging technologies along with the main research gaps in the area of microbiome engineering are identified for incorporation into emerging agricultural practices, such as intercropping systems. Finally, (iv) current scopes and a detailed understanding of the consortia inoculants behavioral response within the plant-soil system are presented, along with the extent to which these inoculants could persist and induce long-lasting effects on subsequent cropping cycles.

### Plant microbe interactions affected by P deficiency and drought stress: a double edged sword for cereals and grain legumes

The frequent occurrence of drought periods is a critical constraint to grain legumes and cereals production causing up to 80% of yield losses worldwide^40,41^. Climate change is predicted to bring about decreased winter rainfall in the range of 15% by 2030 and 30% by 2070^42^ and an increased temperature of about 1.6 °C by 2050. The magnitude of plant production declines caused by the rainfall fluctuating patterns also relies on the soil fertility and varies with the different management techniques, such as fertilization. In fact, the reduced soil water availability due to recurrent drought scenarios typically leads to reduced diffusion rates of soluble nutrients mainly P, resulting in a low P-uptake and build up by the plants^9,43^.

**Fig. 2:**
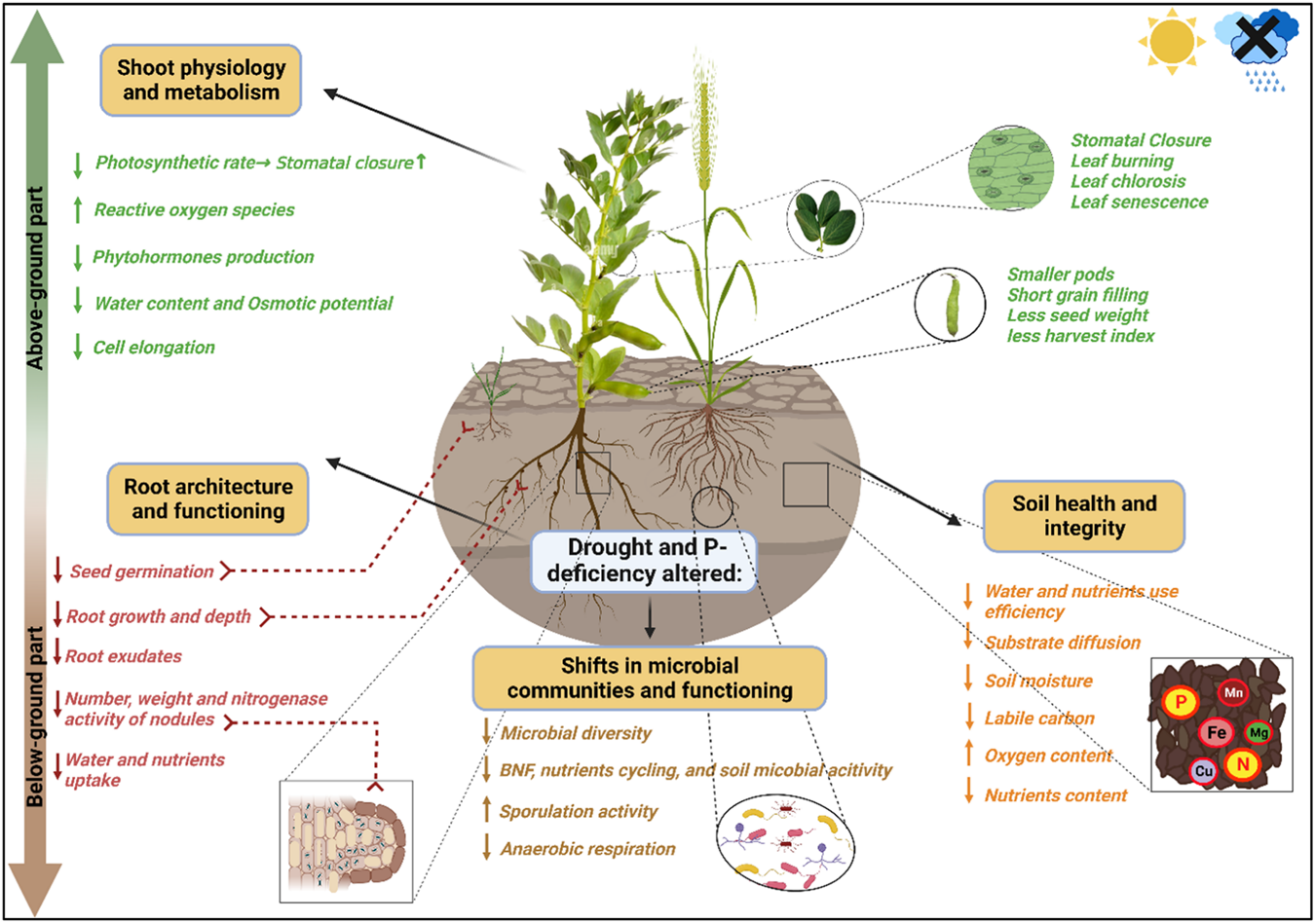
Water and P-deficiency impacts on intercropped plants and root bacteria. Schematic illustration of the effect of water and P-deficiencies on intercropped plants’ above- and below-ground processes. Upward and downward arrows represent the stimulation versus the repression of the plant’s physiological, morphological, and biochemical processes, respectively. Structural and functional diversity of root associated bacteria shows a negative response to both stressors. Of note, both stressors along with root exudates from intercropped species, favor the selection of resilient microbial species. This figure was created using BioRender.com.

Grain legumes like faba bean are highly sensitive to stresses occurring during their growth cycle, especially drought stress that was reported to cause drastic effect on the crops’ growth and yield stability^3,4^. The detrimental effect of drought stress particularly, results from the photooxidative damage caused by ROS to nucleic acids, proteins, membrane lipids and photosynthetic pigments^10,44^. A decrease in photosynthesis, transpiration rate, membrane stability index, gas exchange, and leaf water potential were also noticed in drought and P-stressed, legume and cereal crops^45–47^, ultimately affecting the yield and its related parameters^45^ (Fig. 2). Drought and P deficit were seen to cause poor nodule development and dysfunction in legume crops, which could have negative repercussions on the BNF performance^5^. Given that the symbiotic rhizobacteria relationship is likewise affected by both stresses - drought stress and P deficiency - the reduced nodulation may be an indirect outcome of both effects^3,11^. However, in contrast to grain legumes, research studies stated that cereal crops experience less growth and yield declines as a consequence of the bespoke stresses.

In addition to the drastic physiological and morphological growth alterations, some evidence demonstrates the direct link between cropping systems, soil microbial diversity and environmental constraints^28,48,49^. Indeed, differential responses of soil bacterial community were recorded under intercropping systems, and it was largely dependent on environmental changes. Drought has been reported to affect microbial communities either directly (desiccation) or indirectly (changes in exudates profiles), which further drive shifts in microbial community structure and diversity, consequently, microbial activities^38,50^. As an example, seasonal timing of drought significantly decreased bacterial community diversity and enzymes activities, notably N and P-acquisition enzymes^49^, yet, enzymes activities had mostly recovered, further demonstrating that structural and functional properties are not always synchronized^51^. Under water-deficient conditions, the growth of slow-growing, drought-adapted bacteria, such as Actinobacteria maybe favored^49,52^.

Additionally, soil P status have been proven to drive shifts in rhizosphere microbial community composition and diversity^53^. Under wheat/ faba bean intercropping system, soil P availability was the main driver of the abundance of Proteobacteria, Actinobacteria and Firmicutes^28^. However, opposite trends were noticed in wheat-common bean intercropping as shifts in the root microbial community structure were species- rather than P-driven. More precisely, an increase in bacterial members (e.g., Hyphomicrobiaceae, Bradyrhizobiaceae, Comamonadaceae, and Rhodospirillaceae) associated with enhanced N nutrition of the host plant was noticed, irrespective of the P condition^32^.

Generally, research studies on plant-microbe interactions indicate the enrichment of beneficial bacterial groups able to express specific plant growth promoting functions under drought or P-deficient conditions^54^. Temporal shifts in drought-tolerant PSB functionality and abundance were noticed over sole-cropped wheat growing seasons. In fact, drought tolerance potentials of the dominant species, notably those belonging to *Phyllobacterium*, *Pseudomonas* and *Streptomyces* genera were found to be increased at grain filling stage which coincides with heat seasons^22^. However, despite the ample knowledge of the belowground inter-species interactions and how shifts in root associated microbial communities are driven by crop species and environmental constraints, notably drought and P-deficiency, our understanding of these interactions, specifically in grain legume-cereal intercropping systems exposed to both stressors is still scarce. In fact, most research studies on legume-cereal crops and their associated microbial communities did not focus on studying the impact of drought and P-deficiency on the diversity and composition of the rhizo-microbiome as they rather focused on studying their associated activities. Indeed, drought and P-deficiency led to an optimal soil microbial biomass and activities in a maize-grass pea intercropping system^55^. In contrast to sole-cropped plants, low-P conditions boosted soil phosphatase activity and the acidification process. This tendency helped both intercropped species make better use of the few resources, with drought stress markedly strengthening this interspecific beneficial impact^55^. Similarly, under drought-stressed conditions and available P, microbial community promotion and soil acidification process were associated with enhanced facilitative interaction between maize-grass pea intercropped species. Also, P-deficiency stimulated phosphatase activity which in turn fostered the mineralization of soil organophosphorus, ultimately enhancing P nutrition in both crop species^56^.

Based on the existing knowledge, one could conclude that the dynamic interaction within the soil microbial communities of intercropped plant species, particularly in reasonable legume-cereal based intercropping systems can contribute to ameliorating soil properties to a certain extent. Therefore, understanding the full extent of interaction between plant and associated rhizospheric microorganisms, and how these interactions are affected by drought and P-deficiency should offer new insights on how to improve crop resilience to the bespoke stresses via specific and stress-adapted microbial inoculants.

## Ecological significance of drought tolerant P-solubilizing rhizobacteria in legume-cereal intercropping

Rhizosphere dwellers play a significant role in providing different ecological services, as evidenced through their active participation in a multitude of biological interactions and biogeochemical processes^23^. Rhizobacteria-plant interaction and/ or rhizobacterial consortia-plant interaction may establish relationships to allow a better response to disturbances and adaptation to harsh environmental conditions^20,57^. For example, inoculation of intercropped maize-faba bean with *Rhizobium leguminosarum* (biovar viciae), enhanced the profitability of P fertilizer by increasing P-use efficiency (PUE), nodulation parameters as well as the average grain yields of intercropped plants, especially at low P rates^33^. Moreover, inoculation of the same crop species showed a remarkable increase in plant productivity that was concomitant with a decreased application of N fertilizer rate from 300 to 150 Kg N/ha. This was likely attributed to the enhanced biological nitrogen fixation (BNF) operated by the legume-*Rhizobium* symbiosis^33^.

Interestingly, Inter-, and intra-specific cooperation between microorganisms and intercropped species can boost the advantage of intercropping systems. For instance, a facilitative interaction expressed by an increase in soil fertility in terms of bioavailable P and N, was noticed following co-inoculation of intercropped wheat- faba bean plants with the PSB *Rahnella aquatilis* and *Pseudomonas* sp.^39^. Similarly, co-inoculation of intercropped fenugreek-barley plants with the PSB *Variovorax paradoxus* F310, and *Sinorhizobium meliloti* F42, increased the growth of both intercropped plants at low rainfall regions^58^. Interspecific cooperation between microorganisms may include both bacteria and fungi, as they can be of great benefit to agro-ecosystems by either boosting and/ or complementing each other’s performance. For instance, biofertilization of intercropped maize-soybean plants with arbuscular mycorrhizal fungi AMF (*Rhizophagus irregularis*) and two PGPR (*Streptomyces* sp. and *Bacillus megaterium*) strains strongly increased N and P uptake by both plants under water deficit conditions^59^. In fact, application of microbial consortia could enhance the profitability of P fertilizers by increasing the P availability and uptake efficiency while decreasing the regular recommended P fertilizer rate^31^.

**Fig. 3:**
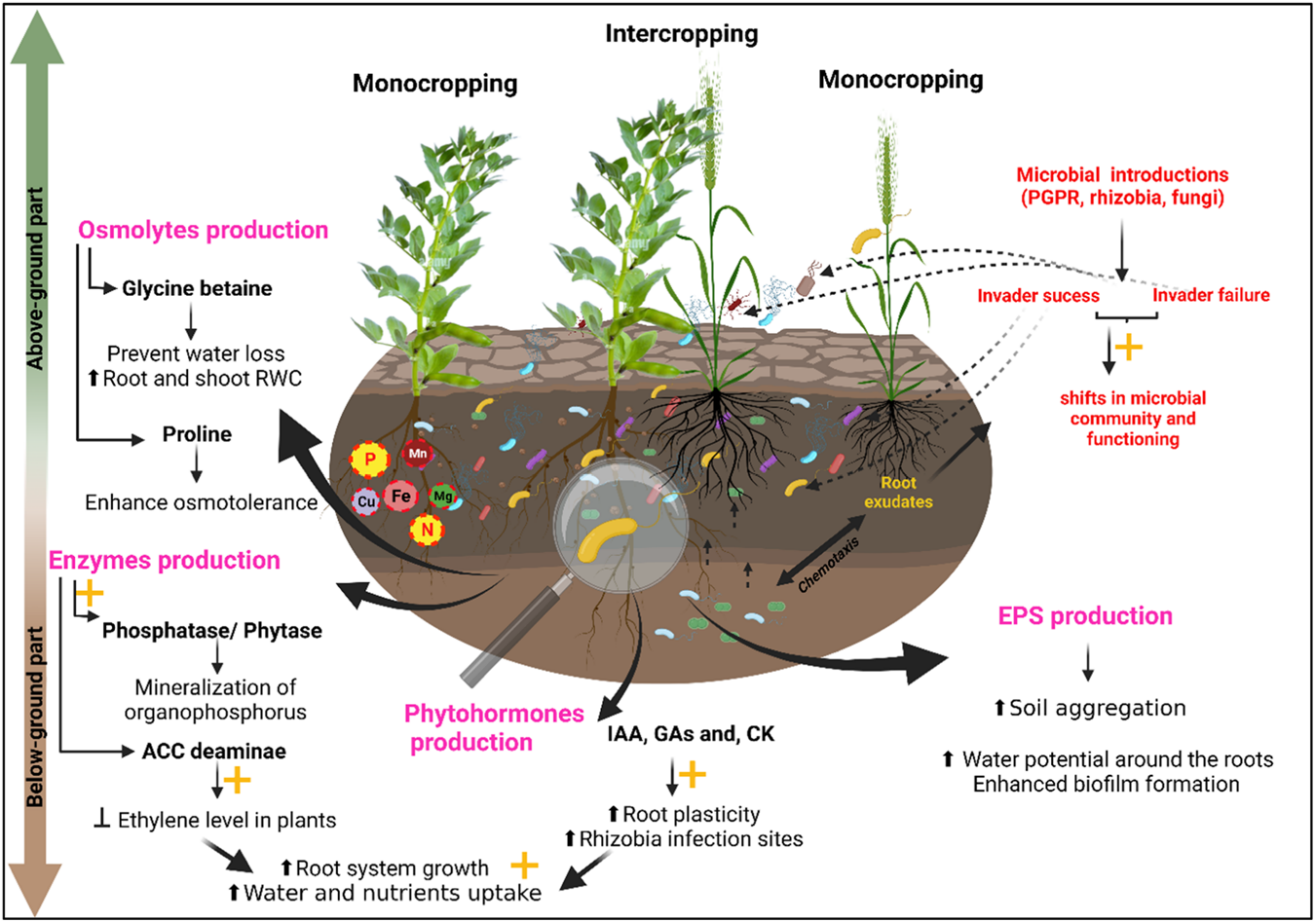
Synergistic effects of microbiome and intercropping on plants. Schematic representation of the benefits of microbiome-based management methods and farming practices (intercropping) on the plant-rhizosphere system functioning. The figure illustrates the beneficial outputs of both management practices on the plant’s physiological, morphological, and biochemical processes. The combination of microbial inoculants and intercropping systems affect positively both above-ground and below-ground crop performance. This figure was created using BioRender.com.

Results from the previously discussed studies shed light on the crucial ecological services provided by the legume-based intercropping system itself. Owing to their lower interspecific interactions with cereals, legumes were reported to increase the N as well as P nutrition of associated cereals, while reducing N inputs and increasing land productivity^34,35^. Therefore, it is worth mentioning that intercropping system cooperates significantly with microbial inoculants to benefit both the plant and the soil (Table 1). However, the effect of co-inoculation with rhizobia and PGPR on intercropped wheat and faba bean under limiting conditions of P and water deficiencies, has not yet been investigated, even though a few studies have mentioned the effect of inoculation on this intercropping system under only one of the previously mentioned stresses. Therefore, further research must be done to learn more about the effect of microbial inoculation on this particular intercropping system in the presence of the bespoke stresses. Studies must also consider the underlying crosstalk between root exudates and rhizobacteria given its major practical response in the establishment of a beneficial root-rhizobacteria interaction that could provide obvious benefit for plant growth and thus decreasing the detrimental effects of environmental stresses. Under intercropping systems, interspecific spatial complementarity between intercrop species, depends on the root architecture and depth and it was found to be involved in a better use of soil resources including nutrients^59–61^ (Fig. 3). Interestingly, there is evidence that under low P^62^ or drought conditions^63^, faster root growth of intercrop species has been noticed following microbial inoculants application. This process was found to be more facilitated by the rich microbial diversity surrounding both intercropped species^32^.

In addition to microbial inoculants, crop stand composition has been found to affect root parameters of plants (Fig. 3). For example, root length, density, mean root diameter, and root dry weight were more enhanced in pea-linseed intercropping compared to pure pea stand or pea-wheat, under *Rhizobium* inoculation^64^. Moreover, cropping system composition, P fertilization as well as, a microbial consortia-inoculant (*R. irregularis*, *Streptomyces* sp. and *B. megaterium*) influenced positively root dry weight of intercropped maize-soybean plants, eventually leading to an enhanced plant growth at 50% of the regular recommended P rate for maize-soybean intercropping^31^. Likewise, inoculation of intercropped pigeon pea - maize plants with *Pseudomonas* sp G22. showed to manifest its effect on root weight and length of both plants, eventually leading to a better growth^27^.

Double inoculation of intercropped common bean and maize with the AMF *Glomus mosseae* and *Rhizobium* was found to increase total and root dry weights of plants. However, unlike previous studies, this positive effect on the root system did not enhance plants tolerance to drought as it was not correlated with a higher stomatal conductance or root water content^65^. Also, a significant decrease was noticed in wheat root surface area when intercropped with alfalfa or soybean plants inoculated with *S. meliloti* or *Bradyrhizobium japonicum*, respectively^66^. According to most of the cited studies, the effect of microbial inoculants in enhancing the root-root interaction of intercropped species have been proved to be significant in stimulating plants’ performance in the presence of abiotic stressors. However, the usefulness of these inoculants and their benefit for crop management in water or P-deficient soils is still debated. The inefficiency of some of these microorganisms may be linked to their preferences for the host plant^27^. Therefore, to further understand the plant-microbe interactions in intercropping systems, research investigations must consider studying multi-strains microbial inoculants and their behavioral response towards host plants.

Although single strain inoculants showed agriculturally valuable functions^20,21^. However, studies under field conditions reported their inability to reproduce laboratory results^67^. In view of this, application of multi-strains consortia as opposed to single strains were found able to provide functional benefits to the host plant and influence inoculants survival^67,68^.

**Fig. 4:**
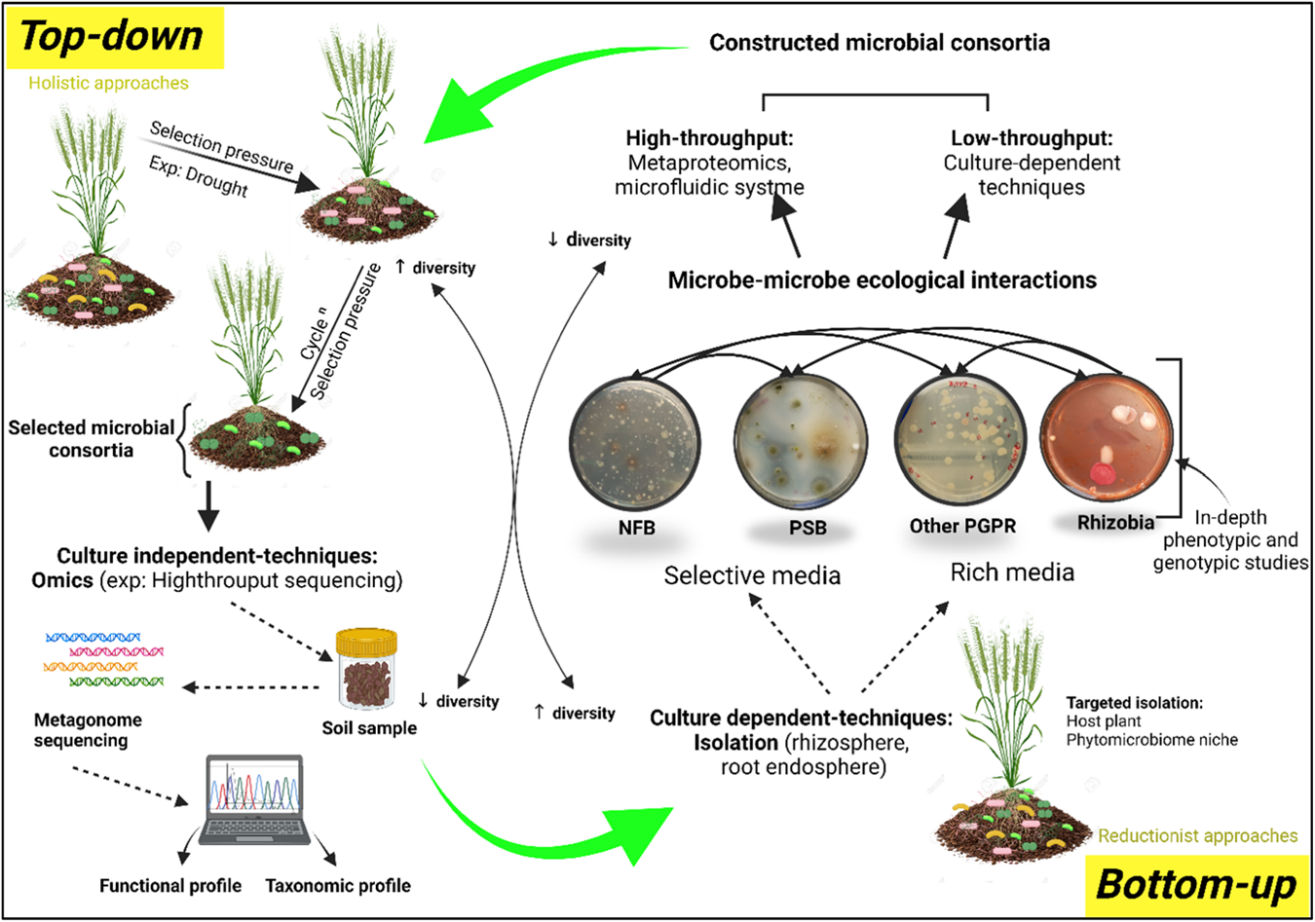
Approaches to microbial consortium construction. Schematic representation of holistic and reductionist consortia construction approaches. The bottom-up strategy allows experimental testing of causality between individual isolates which gives an in depth phenotypic and genotypic comprehension of the microbial behavior either individually or in low-diversity mixtures. Holistic approaches are more likely involved in selecting complex and uncharacterized microbial communities following the application of a particular selection pressure, thus proving a good starting point for isolation of relevant and keystone microbial members for a phenotype of interest. This figure was created using BioRender.com.

Greater likelihood of success by microbial consortia inoculants was found when using compatible microorganisms that cooperate synergistically to enhance plant growth^57,69^. A good example of this, is the cooperative association between rhizobia and other soil rhizobacteria^57,70^. Generally, microbial consortia are constructed following a bottom-up approach using well-defined isolated microbes (Fig. 4) with a large genetic pool that provides optimized downstream impacts^71–73^. Yet, to fully understand the microbial community and functioning, a more holistic, microbiome-based approach supported by next-generation sequencing technologies is required. The below-sections will provide a comprehensive understanding of the microbial consortia construction approaches, drawing from microbial-interspecies interaction to the design of robust microbial consortia capable of reliably enhancing agricultural productivity.

## Understanding the rhizobacteria interspecies interactions: a determinant starting-point towards the construction of rhizobacterial consortia

The binary interaction between plants and their associated microorganisms, particularly, the beneficial rhizosphere dwellers has been extensively investigated^68,74–76^. Yet, the interaction between microorganisms among themselves, especially the one taking place in the proximity of the soil-root interface remains poorly understood. Meanwhile, the extent to which the interspecies interaction shapes the rhizosphere microbial assemblages and functional dynamics are still a matter of discussion^77^. Additionally, the molecular mechanisms underlying behind the ecological inter-microbial interaction, notably, cooperation, resource competition, persistence, and co-existence in microbial mixtures in both, natural environments, or laboratory-controlled conditions, are still not fully explored or still in its infancy^65,67,78,79^. Therefore, there is a considerable need to decipher this intricate interaction at both controlled and natural conditions.

Since their first discovery, the physiology and metabolism of bacteria have been deciphered in rigorous details^70,80^. Of note, in bacterial mixtures, it was found that based on their metabolic rates, only a subset of microbes could show active dynamics while others are likely to experience dormancy. In fact, stochastic changes in the relative abundance and activity of individual microbes within a community result from the intentionally (production of antibiotics) and/ or unintentionally (e.g., resource limitations) unfavorable conditions created either by specific individuals or from the collective metabolic activity of the whole community, respectively^81,82^. In the present date, the emergence of omics-based technologies has led to significant discoveries related to microbial growth and metabolism within an identified or unidentified microbial community^72,82^. Using metaproteomic, the microbial competitive and cooperative metabolic interactions along with the resulted downstream effects have been identified and quantified in large scale^73,83^. For instance, using a reductionist approach coupled with high-resolution mass spectrometry-based metaproteomics individual microbes within a defined community “Defcom” were found able to express genus specific-strategies to adapt with the changing microbial community and environments^82^. In this study, *Pseudomonas*. sp. GM17 was the dominant microbe in the DefCom, this was indicated by its high growth rate, antibiotics, and secondary metabolites production. *Pantoea* sp. YR343 was also able to dominate and resist to the stressful environment created by *Pseudomonas* sp. GM17 mainly through the expression of stress proteins related to carotenoids biosynthesis, motility and antibiotic resistance, as coping mechanisms. Whereas sporulation was the main mechanisms adopted by *Bacillus* sp. to cope with collective stress generated by other community members^82^. Under co-culture conditions, microorganisms could express an altruistic relationship. For example, in the presence of *Enterobacter cloacae* Rs-2, the growth of *B. subtilis* SL-44 was restricted as indicated by the downregulation of genes associated with development and cell differentiation. However, this inhibitory effect has led to the upregulation of genes related to siderophores biosynthesis, thus providing more siderophores to the community^77^.

During intraspecific microbe interaction, the release of metabolites (e.g., DMDS a volatile organic compound “VOCs”) could not only contribute directly to increasing plant growth but could also modulate the expression of plant growth promoting features notably, siderophores production, P, and potassium solubilization^84^. Therefore, we may conclude that bacterial and/ or microbial metabolites, like VOCs, may act as interspecies signaling compound playing an important role in coordinating the expression of plant growth promoting phenotypes. In addition to VOCs, bacterial quorum sensing signaling compounds have been discovered long time ago and were reported to be used by bacteria as to synchronize their metabolic activities^85,86^. Some bacteria have the ability interfere with bacteria-bacteria communication through quenching the quorum sensing activity either through enzymatic degradation of the quorum-sensing molecules, through sequestering them or through interfering with quorum-sensing receptors^86^. In a tri-trophic model *B. subtilis* UD1022 significantly reduced *S. meliloti* biofilm formation through the production of quorum quenching lactonase (YtnP) which interfered with the quorum sensing pathway of *S. meliloti*, eventually having negative effect on growth promotion and nodulation of the host plant^85^.

Most of the studies stated above have demonstrated a positive effect of using low-diversity consortia with several strains ranging from 2 to 10^58,87–89^. Yet, no explanation has been given regarding the rationale behind choosing this diversity. A recent study revealed that reduced-complexity systems, here referred as low-diversity consortia, are a prerequisite for experimentation as a technique to reveal causality and the molecular mechanisms underlying phenotype-genotype relationships through focused manipulation. However, simplified experimental systems cannot mimic natural complexity, therefore, there will be a risk of missing keystone organisms and functions^68^. Taken together, to build a detailed fundamental explanation of the microbe-microbe interaction, research targeting simple subsets of a microbial community, should be evaluated under both, different laboratory, and natural conditions, to predict and further decipher the PGPR functioning in the phytobiome, which aims eventually to produce beneficial synthetic consortia to sustain agriculture under fluctuating environmental conditions.

## PGPR-rhizobia interactions: an advantage in boosting PGP efficiency in intercropping systems under water scarcity and/or P-deficiency

The symbiotic association between legumes and the nodule endophytic bacteria (rhizobia) represent one of the best studied association between plants and their phytomicrobiome^90,91^. Generally, during this symbiosis, photoassimilates produced by leguminous plants are exchanged for the biologically fixed nitrogen by the rhizobia and thus, providing their host with significant amounts of N required for their growth^92^. On the other hand, the beneficial rhizosphere dwellers, also referred to PGPR have been widely exploited for their growth promoting properties such as the solubilization of mineral nutrients, and the production of phytohormones among so many others^93^ (Fig. 3). The cooperative association between *Rhizobium* species and PGPR, enhances plant growth and nodulation parameters in soybean^70^ and common bean^94^ when grown as sole crops in low P soil, and in faba bean and wheat when grown as intercrops^89^. One of the main physiological mechanisms behind this cooperative association is the production of phytohormones, notably auxin, which is known to boost root growth and number of root hairs, the absorption of water and nutrients as well, which all lead to generating more rhizobia-legume interaction sites. Co-inoculation of sole-cropped soybean with IAA- producing *Azospirillum brasilense* Az39 (ipdC+) increased the efficiency of *Bradyrhizobium*-soybean symbiosis, further highlighting the importance of the microbially synthesized IAA in the maintenance of this symbiosis. Similar results have been obtained for common bean when inoculated with the IAA and (1-aminocyclopropane-1-carboxylate (ACC) deaminase.) ACC- producing *Pseudomonas*^80,95^.

According to these studies, we can conclude that a cooperative association may reside when rhizobia and PGPR are co-inoculated into plant roots, given that PGPR are known to affect positively the symbiotic performance of rhizobia^89,94,96^ and enhance nodulation of intercropped leguminous plants in the presence of otherwise abiotic factors. In common bean -fennel intercropping system, the complementarity in terms of growth promotion, that exists between the PSB *Pseudomonas putida* and *Pantoea agglomerans* proved to be efficient in producing phytohormones that stimulate the root traits and increase the P availability, eventually leading to better nodule development and functioning^97^.

In fact, by conferring complementarity benefits, inoculation of intercropped maize and cowpea plants with N fixing microbes (*Rhizobium* and *Azotobacter* spp.), PSB (*P. fluorescens*), and potassium mobilizing bacteria (*B. mucilaginous*), along with different organic and inorganic amendments (e.g., fertilizers and farm-yard manure), enhanced forage quality, production and the land use efficiency under semi-arid conditions^63^. The same bacterial mixture has shown positive effects on forage production and nutrients uptake in maize cowpea intercropping system^63^. It is noteworthy that high concentration of IAA produced by PGPR may sometimes inhibit the legume-Rhizobium symbiosis as seen in intercropped barley and fenugreek inoculated with *S. meliloti* F42 and *V. paradoxus* F310^58,98^.

It is now evident that the application of PGPR along with efficient rhizobial strains can lead to an effective legume-*Rhizobium* symbiosis which ultimately enhances the N nutrition and growth of legume crops^94,99^. Non-nodulated companion species also benefit from the N-fixed by legumes in the case of legume-cereal intercropping system, and this part of N is a crucial source for their growth and development^84,88,89,100,101^. According to the above- described studies, the application of PGPR-rhizobia mixtures in intercropping systems had a significant impact on rhizosphere processes, notably the root exudation patterns by both intercrops, microbial biodiversity and water and nutrients uptake. However, no such study has been so far undertaken to decipher the interactional mechanisms in a *Rhizobium*-PGPR-intercropping interplay in the presence of the combined stresses of drought and P deficiencies, and their subsequent outputs on the growth and the overall production of both intercropped species.

This knowledge paves the way for future studies to decipher the response of legume plants and their intercropped counterparts to the inoculation with *Rhizobium*-PGPR consortia in the presence of abiotic stresses. Understanding a priori the characteristic of each individual strain composing the consortia to select efficient and compatible strains from both bacterial groups (rhizobia and PGPR) is also an important research gap that is worth investigation. This will help selecting multitask bacterial consortia that could satisfy N requirements of both plant species, enhance soil fertility especially in terms of P, and stimulate water absorption under contrasting environmental conditions.

## Engineering drought-tolerant phosphate solubilizing rhizobacterial consortia: perspective into intercropping

Understanding the microbe-microbe interaction and the choice of the appropriate individual strains to form mixed microbial inoculants represents a sensitive and determinant step towards the development of a functional microbial consortia^72,73,85,102^. Therefore, a useful roadmap starting from the isolation and characterization procedures to the design, construction and testing of beneficial bacterial mixtures should be judiciously determined a priori^20^. Generally, microbial consortia are constructed following two main approaches, top-down and bottom-up (Fig. 4). In the top-down approach microbiomes are exposed to selected environmental variables in a controlled manner, through multiple selection cycles, to force the microbiome to gain and/or optimize a particular function coding for a particular plant phenotype through ecological adaptation or evolution^72,73,103–105^. For the bottom-up assemblies, the consortium members, possessing desired growth promoting traits are selected individually from various sources and investigated for their associative interactions using different enrichment or selective culture-dependent methods^103,104^. In recent years, the concept of synthetic microbial communities (known as SynComs), has emerged as a powerful strategy. These communities are designed to mimic specific microbial interactions and functionalities, allowing researchers to study plant-microbe interactions in a controlled and reproducible manner^72,73,95^.

Gathering different microorganisms unifying various growth promoting traits represents the first step towards beneficial synthetic consortia construction in the bottom-up approach^20,106^. This begins with the selection of the appropriate environmental source from which the typical growth promoting microbes should be isolated^89^. Indeed, exploring rhizospheric microbes from niches where they have co-evolved with plants under one or several environmental stresses, might reflect the general behavior of the community^39,58,97,107^. For instance, PGPR isolated from sugarcane rhizosphere grown under drought stress were found to exhibit positive results for one or more plant growth activity and were able to grow under drought stress^106^. Of note, plants are known to orchestrate the chemoattraction of beneficial microbes according to their needs^108^. Therefore, in the presence of abiotic stresses such as drought or P-deficiency, plants tend to change their exudation pattens and exudates composition to favor the selection of beneficial microorganisms, which further reverse the deleterious effects of stressful conditions^25^.

Furthermore, it was recently reported that the type of the cropping system may indirectly have some control over the chemotactic response of the soil rhizobacteria. This is likely due to variation in the exudation pattern and/ or root exudates composition in each system^27,109,110^. For instance, when maize and faba bean grow together, maize root exudates stimulate beneficial bacterial activities such as nodulation and BNF in the intercropped faba bean roots, ultimately enhanced the growth and N nutrition of both intercrops^24,111^. Understanding these intricate interactions between intercropped plants and the rhizosphere microbiota can provide valuable insights into the selection and design of SynComs to support intercropping systems. In intercropping systems under abiotic stresses, SynComs, or synthetic consortia of bacteria, offer promising solutions to improve plant growth and resilience. These tailored microbial consortia consist of specific bacterial strains selected for their growth-promoting traits, which can aid intercropped plants in facing adverse environmental conditions^89,104,112^. For instance, in drought-prone intercropping systems, SynComs containing bacteria that produce indole-3-acetic acid (IAA), such as certain *Bacillus* and *Pseudomonas* species, have demonstrated their ability to enhance water-use efficiency and drought tolerance in intercropped plants^31^. Furthermore, in nutrient-deficient intercropping systems, SynComs composed of phosphate-solubilizing bacteria, like *Streptomyces* and *Bacillus* species, can improve P uptake and overall nutrient acquisition in intercropped plants^31^. In another study, a cross-inoculation method was employed to assess the antagonistic interactions between different bacteria^87^. The experiment revealed that double inoculation with two bacteria (Bacillus sp. and P. putida) led to a significant increase in the root/shoot ratio compared to the control. However, co-inoculation with three or four microbes resulted in lower root/shoot ratios, indicating that co-inoculation with three or four microbes may be more beneficial in promoting aboveground biomass development in plants when required^87^. In fact, literature search revealed that most of the research studies addressed the impact of these factors – abiotic stresses and cropping systems – separately on the plant rhizo-microbiome synchronization^94,97^. However, there is a lack of comprehensive investigations into the impact of SynComs – even though unifying various growth promoting traits – to satisfy plants requirements in the presence of more than one factor, whether it be abiotic stresses, cropping systems, or biotic stresses^94,97,106^. Therefore, further studies need to consider the exploration of microorganisms from intercropping systems subjected to different environmental constraints. This may help to develop specific microbial consortia to improve the effectiveness of intercropping system while decreasing the drawbacks of environmental stresses on crops growth and productivity.

In addition to this, several researchers tended to select isolates showing the highest in vitro and/ or *in planta* growth promoting capacities to formulate microbial consortia. For example, *Azotobacter chroococcum* (AU-1), *B. subtilis* (AU-2), *P. aeruginosa* (AU-3) and *B. pumilis* (AU-4) exhibiting high IAA production, P-solubilization, Fe-chelation and siderophores production were selected to construct efficient microbial consortia^113^. Moreover, the application of a mixture of efficient biofertilizers belonging to different PGPR groups, notably, N-fixing soil bacteria (*A. vinelandii* and *R. phaseoli*), P-solubilizing bacteria (*P. putida* and *P. agglomerans*) and K-solubilizing bacteria (*P. koreensis* and *P. vancouverensis*) increased the advantage of intercropped fennel-common bean crops^97^. Of note, isolates with low PGP potentials should not be excluded when constructing consortia as their low or moderate action can aid efforts to achieve desired outcomes when inoculated to plants along with other synergistic microbes^20^. On the other hand, given that soils are lacking significant levels of native rhizobia, the application of PGPR-rhizobium consortia may improve the efficiency of the legume–rhizobium symbiosis. Consequently, improving BNF process and the growth of legumes and their intercropped counterparts^114^. Therefore, considering the combination of synergistic PGPR and rhizobia strains could be an advantageous strategy to improve the performance of intercropped legumes.

In the context of synthetic microbial communities, synergies among individual members of a consortia are generally evaluated using traditional microbiological methods which relies on co-cultivating different microorganisms in non-selective or selective media which are generally chosen based on the final goal of the screen^57,115,116^. For instance, the assessment of compatibility between PGPR strains (*B. subtilis* and *Paraburkholderia sabiae*) using overlapping growth tests provides insights into their interactions when closely co-cultured. Identifying a compatible interaction, indicative of mutualistic cooperation, highlights the potential for enhanced plant growth and optimized soybean production through improved nutrient uptake and soil conditions^117^. Moreover, a recent study systematically investigated bacteria both individually and in various combinations under saline stress conditions to identify synergistic consortia that exhibit plant growth-promoting traits. Eighteen consortia, encompassing all possible combinations of *Enterobacter*, *Pseudomonas*, and rhizobia strains, were meticulously prepared with equal proportions (10^8^ cfu/ml) of individual strains. Both pure cultures and mixed consortia were thoroughly characterized for their plant growth-promoting traits in the presence of NaCl at varying concentrations (1–5% w/v) to select a consortium which could increase the salt tolerance levels of the plant^57^. This classical method has been used and several consortia were constructed and tested for plant growth promotion under different experimental conditions. However, this technique can only do qualitative analyses, using a limited number of microorganisms, thus it is considered as low throughput^118^. Considering limitations of traditional methods in studying microbial interactions. Co-cultivation on selective/non-selective media may not fully represent natural dynamics. Microbes in nature interact with diverse species and face complex environmental factors not mimicked in labs, affecting observed synergies. Generalizability of findings should be cautious as microbial behavior varies with environmental context (soil, temperature, pH, nutrients), impacting plant growth differently in various conditions.

Meanwhile, with the development of recent advances in multi-omics and high-throughput technologies, new strategies for studying microbiological networks and interactions among the synthetic community members have been emerged as promising tools to profoundly understand and select microbiomes with desired functions^119–121^. Microfluidic culture system helped to screen antagonistic or growth promoting interactions between a focal species (*Pantoea* sp. YR343) and a distinct random sample of uncharacterized microbes from plant rhizosphere^122^. The combination of flux balance analysis accompanied with growth curve analyses, and meta-proteomics helped in providing fundamental knowledge regarding the metabolic interactions among plant-associated microorganisms and which could eventually help in designing microbial consortia with desired biological functions^104^.

Although constructing diverse consortia of strains carrying redundant or complementary functions holds promise, yet it is generally unknown how such consortia would establish across a variety of environments and whether their establishment might influence crops growth over growing seasons. Indeed, the knowledge described in this review demonstrates that microbial inoculants frequently lack ecologically relevant traits that would allow them to both survive in the field and provide a legacy effect over growing seasons. Instead, they are frequently selected based on their activity in controlled laboratory screening experiments and ease of mass cultivation^67^. Therefore, studies focusing on the selection of efficient microbial inoculants that would persist and provide long lasting effect for subsequent crops’ growth represent an interesting theme that urgently needs to be considered in future research.

## Direct and indirect effects of soil microbial legacy on plant growth and soil functioning

At field scale, the capacity of microbial inoculants depends primarily on their ability to adapt to environmental changes to persist and compete with soil indigenous microbiome^93,95,123,124^. However, the extent to which microbial inoculants could establish and persist as to provide long lasting effects on the plant-soil system is a current knowledge gap that needs to be further deciphered^125,126^.

In fact, PGPR legacy occurs through two main mechanisms: augmentation and displacement during which the introduced PGPR could either co-exist with resident soil microbiome while maintaining an unchanged taxonomic diversity and variable function and abundance, or, by changing the resident microbiome abundance and frequency^94,127,128^. It is generally believed that displacement is the most frequently mechanism used by PGPR to persist within resident community, and the retained introduced microbes can leave a legacy effect following the process of niche construction, leading to further changes in community^95,124,129,130^.

Despite the dearth in research regarding the cascading legacies of microbial inoculants in different cropping systems (e.g., crop rotation or intercropping systems), a few studies stated that PGPR inoculation might generate lasting effects that could continuously benefit plant growth particularly by modulating (interactions in the root zone) plant root metabolism at an early growth stage^104,125,126,131–133^ (Table 2). Accordingly, interactions at the root zone, especially under intercropping systems was found to induce legacy effects, indirectly, through changes in the rhizosphere microbial communities^87,97,104,134,135^. For example, a facilitative interaction between intercropped maize- faba-bean and wheat-faba bean drove shifts in rhizosphere microbial communities as they favored the selection of beneficial microbes, for instance, *Rhizobium*, which eventually led to overyielding of subsequent intercropped plants relative to sole-cropped^104^. Indeed, a soil microbiome-mediated advantage sustained the benefits of wheat and faba bean intercropping compared to sole-cropping when grown on a soil from of a previously established experiment^136^. Similarly, maize-grass pea intercropping system left an effect on soil N processes (mineralization and nitrification), which was mediated via the beneficial soil communities in the intercropping system^134^.

**Table 2:** A list of studies reporting the legacy effect of microbial inoculants.

Crop species	Cropping system	PGPR Strains	Inoculation stage	Persistence duration	Production system	Effects on plants	References
Pepper	Monoculture	*Bacillus velezensis* NJAU-Z9	Pre-inoculation of seedlings	Two season trial legacy throughout growth stages	Field experiment	Higher rhizosphere bacterial richness which resulted in plant growth promotion and steady yield enhancement	^139^
Faba bean and potato	Rotation	*Rhizobium gallicum* 8a3 and/ or *Ensifer meliloti* 4H41	Pre-inoculation of faba bean seedlings at two leaf stage	March-June 2009 for faba bean September-December 2009 for potato grown in rotation	Field experiment	Rhizobial strains stimulated faba bean growth and enhanced its rotational effect in potato cropping systems by providing fixed N, increasing microbial diversity which all enhance its growth and resistance to diseases	^131^
Faba bean	Monoculture	*Agrobacterium* sp. 10C2	Sowing seeds in soils previously inoculated for 15 days	80 days post-soil inoculation	Greenhouse experiment	Inoculation increased nodule number, plant biomass, as well as grains’ P, polyphenols, flavonoids and total antioxidant capacity. richness and structure of potential bacterial phyla were positively induced.	^145^
Oat and alfalfa	Rotation	*Sinorhizobium meliloti* and/ or *Azotobacter* spp., *Pseudomonas* sp., *Enterobacter* sp., *Bacillus megaterium*	–	–	Greenhouse experiment	Promoted alfalfa growth, yield and N-accumulation through the inoculation of a preceding oat crop	^146^
Ryegrass and alfalfa	Rotation	*Sinorhizobium meliloti L3Si*	Inoculation of sown reygrass seeds	6 weeks for reygrass in 2009 6 weeks for alfalfa in 2010	Greenhouse experiment	Inoculation enhanced growth of both plants and increased rhizobial abundance, nodulation, and total N-uptake of subsequent alfalfa planst	^147^
Maize and wheat	Monoculture	*Bacillus sp*	Maize seed coated with the PSB	Between 5 June to 10 September 2016 for maize and fall of 2016–2017 for wheat	Field experiment	Inoculation improved grain yield, soil properties, RP solubility and P-uptake in both plants	^93^
Alfalfa - wild rye	Intercropping	*Sinorhizobium meliloti*	Inoculation of sown seeds	Two years experiment from 2006 to 2008	Field experiment	Inoculation induced remarkable shifts of the rhizosphere bacterial communities, and enzyme activities after and enhanced yield parameters	^142^

To this end, the positive legacy benefits of soil microbial communities driven by different cropping patterns, particularly intercropping, have been well documented^137^. However, an unresolved question is how the plant- and/ or the microbially-mediated legacies may respond to abiotic stresses, notably drought and P-deficiency. According to the previously discussed studies, and from an agricultural perspective, the way the soil is manipulated was found to greatly influence the growth of subsequent crops. Therefore, the development of suitable microbial consortia that could establish within the soil and withstand the fluctuating environmental conditions may induce lasting effects throughout multiple cropping cycles.

Moreover, the agro-ecological functions and services provided by PGPR introduction can be driven indirectly through their effect on diversity and composition of rhizosphere indigenous microbiota^94,138^, and sometimes this effect could even reach bulk soil microbial communities^131^. The cascading effects of these microorganisms depend on the inoculant type and richness, in case of microbial consortia^87,96,100,104,111,131^. For example, changes in resident microbiomes have been observed in potato^131^, tomato^111^, and pepper^139^ plants inoculated with *R. gallicum* 8a3 and *Ensifer meliloti* 4H41, *Pseudomonas* or with *Bacillus velezensis* NJAU-Z9, respectively. Cropping systems in concert with microbial inoculants could also influence the structural and functional diversity of microbes throughout continuous cropping cycles^140,141^. For example, the inoculation of alfalfa (*Medicago sativa* L.) - Siberian wild rye (*Elymus sibiricus* L.) using *S. meliloti* induced remarkable shifts of the rhizosphere bacterial communities, and enzyme activities after two years, which was positively reflected on plants’ yield^142^. Similarly, during two rotations of vegetable crops (okra, pea and cowpea), culturable microbial diversity as well as total and functional microbial diversity – assessed by Denaturing Gradient Gel Electrophoresis – increased following inoculation with a combination of AMF and pseudomonads^141^. Of note, unsuccessful invasions by PGPR introductions may still leave a footprint on the soil microbial community and functioning^74,115^. In a few newly published studies^143,144^, *Escherichia coli* was used as an unsuccessful invader to evaluate its effect on native microbial communities. Results from these studies revealed that although being transient and unsuccessful, *E. coli* was found to compete with resident species for resources, subsequently modifying the diversity, niche breadth and functionality of soil communities. Thus, may cause a tangible legacy effect that could likely influence future invasion attempts^143,144^.

## Effect of one-off inoculation on cereals grown in succession (rotation)/or intercropped to legumes

Research studies reported that microbial inoculants or microorganisms associated with previous crops could influence the growth of subsequent crops either directly, or indirectly by leaving a footprint on resident microbial communities^104,143^. For example, fifteen days post-inoculation, *Agrobacterium* sp. 10C2 affected only community structure in non-planted soils. Whereas it positively induced TRF” Terminal Restriction Fragment” richness and structure of potential bacterial phyla^145^. Thus, it can be presumed that beneficial effects of microbial inoculations on crop rotations is not only due to the direct and indirect PGP effects carried by these inocula, but rather due to the stimulation of beneficial bacterial populations.

Recent, studies evaluating the residual effect of microbial inoculants in crop rotation systems have shown that inoculation of preceding crops, with PGPR, may have some control over the establishment of beneficial bacterial populations in the rhizosphere of subsequent crops^92,93^ (Table 2). Moreover, other studies along these lines have demonstrated that microbial inoculants effect on subsequent crops might be specific to certain strains. For instance, alfalfa growth, yield, and N-accumulation were promoted through the inoculation of a preceding oat crop with *B. megaterium* and *Azotobacter* spp.^146^. In addition, a two-season field cultivation of pepper seedlings previously grown in a bio-nursery substrate containing *Bacillus velezensis* NJAU-Z9 significantly promoted plant growth and led to a steady yield, thus demonstrating an induced legacy effect throughout growth stages^139^. Similarly, in a crop rotation system, inoculation of common bean with two indigenous rhizobia strains induced significant changes in microbial communities at the end of the crop season, depending yet on the inoculant type. The extent of these effects marked also the subsequent potato cropping system, in more ways than just furnishing nutrients, but also through plant growth stimulation, enhancing disease resistance, or also by improving the plant’s ability to withstand abiotic stresses^131^. Interestingly, inoculating preceding ryegrass plant system with *S. meliloti* strain L3Si enhanced plant growth and increased rhizobial abundance, which also benefited the subsequent cropping system of alfalfa.^147^. This has led to an abundant nodulation along with a significant increase in growth and total N uptake of alfalfa plants^147^. Inoculating maize with the PSB *Bacillus* sp. and AMF induced lasting effect which resulted in the enhancement of wheat growth and yield, as a subsequent crop^93^.

Unlike these studies, pre-inoculation of wheat with *B. japonicum* did not benefit the growth of subsequent soybean plants^92^. Also, the beneficial effect of N-fixing bacteria (*B. megaterium* W17, *P. fluorescens* W12, *A. chroococcum* HKN-5, and *A. brasilense* CW903) on *Cyclocarya paliurus* could not be maintained without periodic inoculation. Thus, we could conclude that plants respond differently to microbial inoculants which could influence the durability of their beneficial effects. On the other hand, microbial inoculants, based on their type, invasiveness, carried function, richness, and inoculation time could help plant orchestrate the recruitment of specific microbial communities that could potentially stimulate plant growth, likely inducing a legacy effect (Table 2).

Moreover, most of the cited studies used molecular techniques involved in analyzing DNA fragments (TRFLP) and quantifying specific DNA sequences (qPCR) to understand and compare the changes in microbial communities in soil samples, at the end of each crop season^95,111,141^. Traditionally, this impact has been studied by looking at changes in the diversity and abundance of the microbial communities, essentially what types of microbes are present^138^. Yet, it is important to investigate how microbial inoculants affect the soil community’s function^138^. To achieve this, several multi-omics techniques (e.g., metagenomics, meta-transcriptomics, metabolomics, and meta-proteomics) could be used to understand whether the microbial communities, even when changed by the introduction of inoculants, still perform their essential functions^115^.

## Conclusions and perspectives

Single or composite effects of environmental factors impact the fitness and performance of both, the plants, and the soil-dwelling microorganisms. Thus, emphasizing the need of analyzing interactions within the plant-rhizosphere soil continuum. Microbiome-based soil management notably legume-cereal intercropping systems, holds great promise in sustaining plants’ growth, stress tolerance, and the promotion of soil ecosystem functioning and health. Most of the studies discussed in this review highlight the advantage of using multi-strain microbial consortia owing to the variety of outcomes they can provide. Indeed, in advanced agricultural research, multi-strains microbial inoculants are gaining much attention, as shifting from single specific microbes to more diverse microbial inoculants are likely more efficient in stimulating crops agricultural outputs as well as soil ecosystem functioning.

On the other hand, beneficial legume-cereal intercropping systems, are nowadays well known for their high productivity relative to sole-cropping. This is likely attributed to the shifts in rhizospheric microbial communities driven by intercropped species. However, most of the studies addressing the impact of intercropping system in concert with microbial inoculants on plant growth and soil microbial communities have been done under controlled conditions. This may overestimate the beneficial effects of these practices while neglecting the effects of environmental factors on the reciprocal interaction between plants and their associated microorganisms as well as on the durability of their effects. Moreover, the residual effect of microbial inoculants on multiple cropping cycles is still up to debate as many studies focused on evaluating the microbially-mediated growth promoting phenotypes for a short period, yet it remains uncertain whether subsequent crops benefited from these effects. Therefore, we urge future research to fill these gaps as it is crucial to predict the ecological significance of microbial inoculant, plant diversity and their subsequent trajectory in the plant-soil system. With this in mind, we advocate for further studies to address the research gaps proposed below:

- New approaches to consortia construction starting from isolation and screening to the final formulation step should be determined, a priori, while considering the diversity and compatibility amongst consortia individuals. Applied research efforts in this direction will help us to design and produce efficient multi-strain inoculants that could reflect reproducible results on both above- or below-ground crop performance under controlled and field conditions. This should also consider the capacity of microbial inoculants to establish and persist within indigenous soil microbial communities and throughout multiple cropping cycles.
- To maximize profit of microbial biofertilizers, further attention should be oriented to study the legacy effect of microbial inoculants under different cropping systems and in the presence of different environmental conditions that represents an ultimate threat to crops growth and production, for instance drought and nutrients deficiency to build a sustainable next generation agriculture.
- As microbial legacies can be under the control of various factors, we recommend further studies that attempt to unravel the interrelationship between all these factors – environmental stresses, cropping system, and microbial consortia - and their possible outputs in a specific system (e.g., intercropping system), as this would help developing promising inoculants with the possibility to resist to stresses as to induce lasting effects.
- Studying the combined effect of intercropping systems along with efficient microbial consortia will provide a necessary background for the successful application of biofertilizers and to determine the degree to which the later would contribute to enhancing the advantage of intercropping systems.
